# The Medical School's Entry in War Time

**Published:** 1916-09-02

**Authors:** 


### The Hospital

The Workers' Newspaper of Administrative Medicine and Institutional Life, Administration, National Insurance and Health.

No. 1578, Vol. LX. SATURDAY, SEPTEMBER 2, 1916. PRICE ONE PENNY.

### THE MEDICAL SCHOOL'S ENTRY IN WAR TIME.

Those who have longest been associated with the active administration of a medical school attached to a voluntary hospital in this country will agree that there is no time so full of interest, and even excitement, in the case of competing schools as the first fourteen days in October in each year. It is within our experience that parents very often make it their business to visit particular hospitals, to interview the school authorities, sometimes to visit the school buildings, museums, and so forth, and, after devoting hours or days to the business, ultimately to make up their minds where and when the young hopeful shall enter on his career as a medical student. The successful dean of a medical school and his helpers, in proportion to the experience they have had, make it their business to shepherd the parents, to study and even to treat them psychologically, and the result, so far as the entry is concerned, has been governed within our knowledge to a great extent by the personal charm and persuasive personality of the school's representative. Cheerfulness, gracious consideration, and an infinite capacity for taking pains, combined with a knowledge of human nature and the points to which most parents attach primary importance, are amongst the essentials to complete success.

The dean of every medical school will probably have an entirely new experience in regard to the entry of students in October 1916. The war has revolutionised old conditions and minimised many of the chief attractions at most medical schools throughout the country, owing to the depletion of the medical staff and the absence of many leading lights at the Front or in military hospitals where the work keeps them constantly occupied. What policy is best calculated to produce the maximum result so far as the entry is concerned in such conditions? Clearly one which is based upon the war, the putting forward of special and novel points of interest created by the war in regard to hospitals, which cannot fail to arouse the interest and fix the attention not only of the parents but of the students. The field for such observation created by the war should prove fruitful to the dean of resource. In most hospitals there will be the wards in which war patients are under treatment, and in some accommodation for officers, some of whom may possibly be known to a visitor, a point not to be overlooked in the interview with him or her in the medical school office. Here occasion may be taken casually to mention some of the wonderful results achieved by modern surgery and treatment, and the rapidity of the recoveries which have resulted from treatment. What would the country have done without the co-operation of our great voluntary hospitals? And what would have been the fate of the wounded had it not been for the existence of our medical schools, their able teaching and the noble traditions which they have built up and taught to the younger generation of medical practitioners? A casual visit to the museum under knowledgeable guidance can be made full of interest, and we have known it, when backed by a few telling words as to the importance of pathology and its bearings upon success in practice and diagnosis, to create such an impression that a return to the medical school office has been suggested in order that the student may be entered forthwith.

There are, of course, parents and parents. They naturally have to consider, and do consider, finance in its twofold aspect of the amount of fees a student has to pay, and tht prospects of a return on this capital outlay when he receives his diploma and enters upon practice. There have been few changes in the matter of fees recently, so we can concentrate our attention upon the yield from medical practice on the part of the average practitioner who has steadily worked throughout his college days and availed himself to the full of the opportunities afforded him of obtaining practical knowledge in every branch of the profession. Here a few words of wisdom may be the means of impressing the parent with the feeling that their son or daughter will be safe under the wise and fatherly guidance of the affable dean and his colleagues.

In our view, therefore, the year 1916 may be made a record entry by every medical school where its representatives use the war to enforce two points. First, that the resulting wastage in the profession must increase the demand for and raise the standard of medical practitioners in years to come. Secondly, that at no time in its history has the medical profession had such an opportunity to become admirably organised and equipped as it has to-day or capable of offering so great an opportunity to the ablest men of the nation to join its ranks and win a high reputation for knowledge and skill, with the gratitude of an ever-increasing number of patients. Man has made the implements of war more terrible than ever before, but the medical man has had the proud privilege of making the lot of the wounded and sick warriors and their chances of recovery infinitely better and infinitely greater than would have been believed possible two years ago. Is there any profession open to youth which offers greater interest or so absorbing an occupation as the medical profession does in 1916?

